# The burden of size and growth for the juveniles of large mammalian herbivores: Structural and functional constraints in the feeding biology of juveniles relative to adults in red kangaroos, *Osphranter rufus*

**DOI:** 10.1002/ece3.7750

**Published:** 2021-06-14

**Authors:** Terence J. Dawson, Melinda A. Norton, Suzette Rodoreda, Sarah K. Abbott, Steven R. McLeod

**Affiliations:** ^1^ School of Biological, Earth and Environmental Sciences University of New South Wales Sydney NSW Australia; ^2^ Fowlers Gap Arid Zone Research Station Fowlers Gap NSW Australia; ^3^ NSW Department of Primary Industries Vertebrate Pest Research Unit Orange NSW Australia

**Keywords:** cost of mammalian growth, developmental allometry, diet and age in kangaroos, foregut fermentation, kangaroo gut structure, kangaroo population structure, rates of digesta passage, skull and teeth growth

## Abstract

1. Juvenile mammals in their postweaning developmental stages face many challenges in transitioning to adulthood. Among large grazing species such as ruminant bovids and cervids, an overarching challenge is acquiring and processing sufficient nutrients to survive and grow, with a gut that may not yet be fully developed. Marsupial kangaroos of Australia face similar challenges; they also digest vegetation by fermentation in a large foregut. In red kangaroos, *Osphranter rufus* (=*Macropus rufus*), the dominant species of Australia's arid interior, females may breed continuously; however, juvenile recruitment to the adult population is irregular and coincident with sporadic rainfall.
2. As compared with adult females, the nutritional requirements of juvenile *O. rufus* are high in relation to their body mass (BM), largely due to the cost of their rapid growth. We examined processes that juveniles have in their morphology, physiology, and behaviors to meet their elevated nutritional needs, by comparing recently weaned juveniles of both sexes and adult female *O. rufus* in their desert habitat. Features studied include relative body sizes, relative dimensions, and capacities of principal gut regions, the foregut, small intestine, caecum, and large intestine with rectum. Also examined were digesta attributes and rates of digesta excretion. Additionally, the rates of change in skull parameters and dental characteristics to maturity were assessed. Field determinations of diet choice were made for both age classes.
3. In juveniles, the content masses of major gut structures were related to body mass (BM), as were those of adult females, that is, ~BM^1.0^. In both age classes, the digesta mass of the foreguts exceeded 75% of the total digesta mass. Diets of both juvenile and adult *O. rufus* largely focused on grasses. Juveniles had higher rates of digesta excretion while foraging than adults. In addition, the foregut contents in juveniles occupy proportionally less of the total gut than in adult females. Together, the higher excretion rate and smaller relative foregut of juveniles suggest that they necessarily focus on forage that can be rapidly digested, such as young, green grasses, or herbage.
4. Comparison of the skulls of juveniles and adults revealed how this harvest can occur. Relative to BM, juveniles had skulls of larger volume than adults. Additionally, during growth the skull lengthens proportionally faster than increasing BM. By weaning, the dimensions of the incisor bite of juveniles neared those of adult females. The area of wear on premolars/molars increased only slowly relative to the development of incisors, further pointing to juveniles selecting more digestible forage than adults. The intermittent availability of such forage, principally young grasses, appears key to the significant recruitment into the *O. rufus* population in their arid habitat.

## Graphical abstract

The cost of rapid growth impedes the survival to maturity in young grazing kangaroos, particularly for the arid inhabiting red kangaroo, *Osphranter rufus*. Use of a foregut fermenting digestive system by these marsupials constrains gut adjustments, but patterns of skull and teeth growth, combined with marked diet selectivity, enable increased forage harvesting and processing by juveniles.

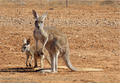

## 1 INTRODUCTION

**FIGURE 1:**
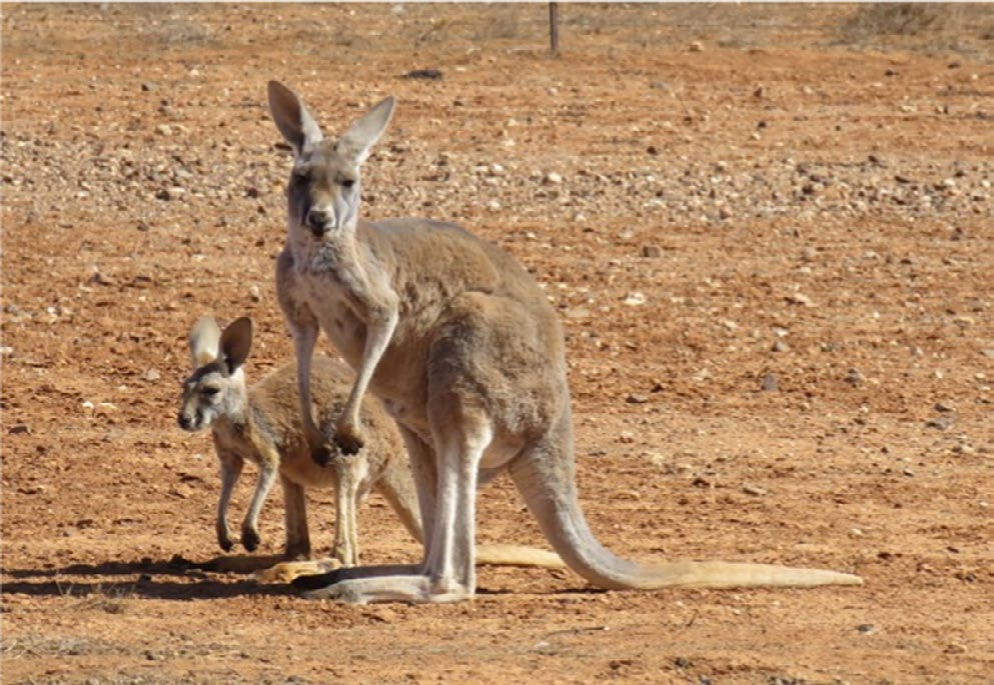
Red kangaroo (*Osphranter rufus*) female with weaned young at foot in the arid inland of Australia. Females breed continuously but such growing juveniles face nutritional challenges in this unpredictable environment. However, relative to adults, they show modifications in their morphology, physiology, and behaviors that facilitate survival to maturity in less severe seasons

Juvenile mammals in their postweaning developmental stages face many challenges in transitioning to adulthood. These include high predation, nutrient deficiencies, behavioral naivety, and thermoregulatory challenges. Marsupial kangaroos, the apparent ecological analogues of ruminant ungulates, also digest vegetation by fermentation in a large foregut, but one that shows less complexity than the rumen (Dawson, 2012; Hume, 1999). Red kangaroos, *Osphranter rufus* (=*Macropus rufus*, Jackson & Groves, 2015), the dominant kangaroo species inhabiting Australia's arid interior were the focus of the present study (Figure 1). As with ungulates, the overarching challenge to *O. rufus* juveniles in their progress to maturity is acquiring and processing sufficient nutrients to survive and grow (Gaillard et al., 1998, 2000; Newsome et al., 1967; Owen‐Smith et al., 2005; Saether, 1997). While fermentative digestion allows access to nutritional resources in fibrous vegetation, and therefore provides a wider dietary niche for these specialist herbivores, what are its nutritional implications for small juvenile *O. rufus* in their erratic, arid habitat?

Allometric analyses relating to body mass suggest that among adult mammalian herbivores, the wet matter (WM) content in the gastrointestinal tract (GIT) varies directly with body mass (BM), that is, BM^1^, and energy requirements and intakes scale to BM^0.75^. Thus, larger mammals putatively have a higher GIT capacity per unit of ingested food and, consequently, a greater digesta retention capability. Such relationships have been used in nutritional and ecological modeling (Clauss et al., 2013; Müller et al., 2013); however, Clauss et al. (2013) caution use of such procedures because of marked variability in the morphology and ecology of species from which allometric relationships are often derived. Predictions resulting from these relationships (Demment & Van Soest, 1985; Parra, 1978) suggest that for adequate, foregut fiber digestion in adult placentals, BM should be above 9–15 kg.

The significance of this for *O. rufus* juveniles is unclear. Though they have barely reached this BM at weaning, laboratory trials show more extensive nutritional capabilities (Munn & Dawson, 2003a, 2003b, 2006). When fed good quality hay, they grew optimally and had digestible energy intakes (DEI) that were 93% of adult female intakes despite their BM being only 38% of adult female BM. These relatively high juvenile DEIs are due to the nutritional demands of growth, that is, the considerable cost of laying on new tissue, plus a small allometric effect. The limits on the ability of juveniles to meet their high nutritional requirements are seen, however, in their response to variation of feed quality (Munn & Dawson, 2006). A 50% increase in dietary fiber content decreased feed digestibility from ~60% to ~44% in both weaned and mature individuals. The juveniles did not sustain growth and additionally lost BM. However, adults did maintain BM on this diet. They had reserve GIT capacity, in which the juveniles did not, and adjusted GIT fill and rates of passage of digesta so as to maintain DEI.

Curiously, in their arid, seasonally unpredictable environment female *O. rufus* mostly breed continuously, but with marked fluctuations in juvenile survival, with mortality commonly occurring around weaning (Frith & Sharman, 1964; Newsome, 1965; Newsome et al., 1967). Population maintenance has been shown to be predicated on substantial recruitment during infrequent, protracted wet seasons (Newsome et al., 1967). Newsome (1966) regarded both diet quantity and diet quality as impacting in these recruitment events; other observations concur (e.g., Moss & Croft, 1999) but the ecophysiological processes involved are uncertain. Weaned, juvenile *O. rufus* reach maturity by overcoming the constraints of small BM through effectively utilizing the sparse vegetation of their habitat; however, this occurs with differing success across varying seasons (Frith & Sharman, 1964).

The present study examines, the ontogenetic changes in morphology, physiology, and behaviors available to juveniles to moderate the challenges faced in their transition to maturity in their desert environment. To do so, we compared recently weaned juveniles and adult female *O. rufus* in this habitat. Features studied include relative body sizes, relative GIT dimensions, and capacities of principal gut regions, the foregut, small intestine, caecum, and large intestine with rectum. Also examined were digesta attributes and rates of digesta excretion. Additionally, since a kangaroo's mouth is its forage harvesting apparatus and its characteristics will impact on diet quantity and quality, the rates of change in skull parameters and dental characteristics to maturity were assessed. Field determinations of diet choice were made for both age classes.

## 2 MATERIALS AND METHODS

### 2.1 Study site

Fowlers Gap is the Arid Zone Research Station of UNSW Sydney. It lies in desert rangeland in the northwest of the State of New South Wales (31.4°S, 142.7°E). This 40,000 ha station is typically vegetated with halophytic, perennial shrubs (Family Chenopodiaceae) and perennial grasses such as the tall *Astrebla* spp. and medium *Enneapogon* spp. Rainfall is low and variable; over a 50‐years period encompassing our study, it was 239 ± 100 mm (yearly mean ± *SD*). Annual plants, largely grasses and forbs (herbage), are abundant following erratic significant rainfall. Our work occurred in two phases. In 1995, data were collected for a study of nutritional attributes in naturally foraging juvenile *O. rufus* as compared with adult females. The second phase, in 2001, collected data to more closely study their natural foraging. This utilized habituated animals in naturally vegetated enclosures and focused on the differential rates digesta excretion and diet selection. The overall study took and held kangaroos under the provision of licenses (A18 and 10194) from the NSW National Parks and Wildlife Service. The UNSW Animal Care and Ethics Committee approved this project, ACEC (95/53, 99/18).

### 2.2 Study animals, body size, and dimensions

This study examined functional features relating to different age classes of red kangaroos feeding in the wild. It investigated the structure, dimensions, and contents of the GIT of recently weaned young‐at‐foot *O. rufus* for comparison with data from mature adult females. For this, a sample of 22 animals was obtained from the Station's Hotel paddock during 13–24 February 1995. This occurred between 06:30 and 09:00 hr, the time when animals were finishing nightly foraging (Watson & Dawson, 1993). Ten juveniles (five males and five females) and twelve adult females were taken by an experienced, licensed shooter. Though *O. rufus* is notably sexually dimorphic, the divergence in BM between sexes commences beyond weaning (Dawson, 2012). Pairs of mother and juvenile were taken, where feasible, to enhance the tightness of comparative data, additionally, to also ensure that lactation had fully ceased from the teat pertinent to the collected juvenile. This was the case, but of note, following the usual reproductive cycles of *O. rufus*, new, small young were often present in the pouch; median size was 364 g, *N* = 10. The metabolic impost on female macropodids of such early‐stage pouch young is low (Baudinette et al., 1988; Hulbert, 1988).

Body mass was determined to 0.05 kg using a Salter 50 kg hanging balance. External body dimensions such as leg, arm, and body lengths were measured to 1 mm with flexible measuring tapes. Juvenile and adult female individuals were initially aged according to the arm/foot relationships (Dawson, 2012; Edwards et al., 1994). Animals were re‐aged later using more reliable estimates via molar progression measures on skulls (Kirkpatrick, 1965).

### 2.3 Gastrointestinal morphology

**FIGURE 2:**
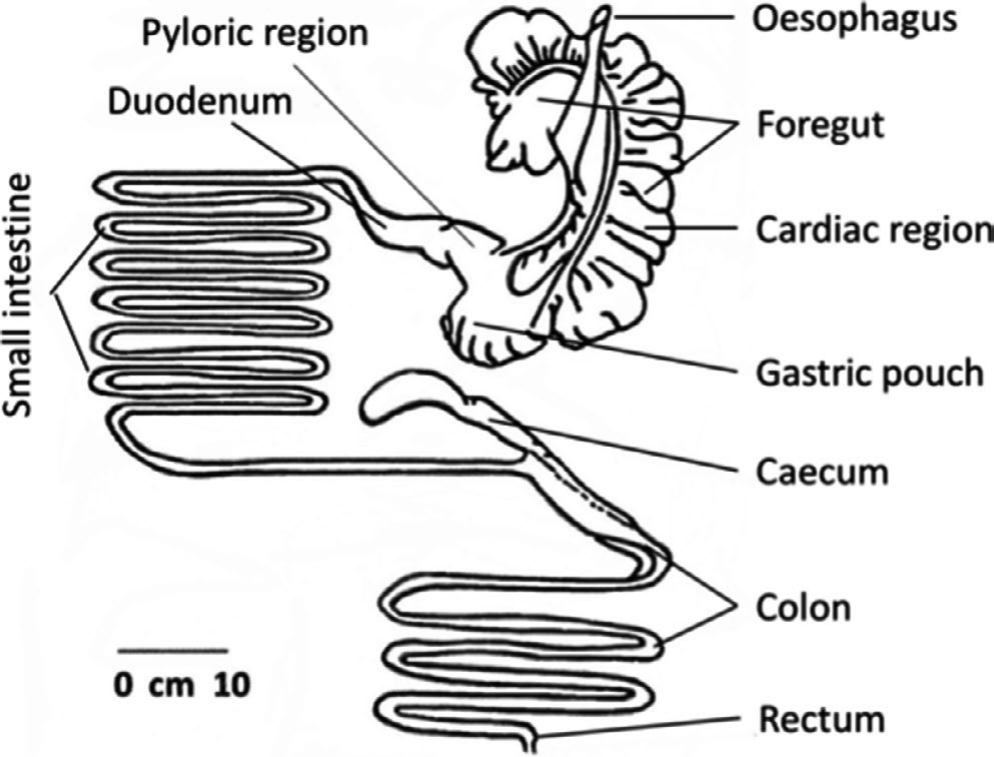
Gastrointestinal tract of an adult female red kangaroo, *Osphranter rufus*. It is drawn approximately to scale using our measurements (Table 2)

As with other species in the Family Macropodidae*, O. rufus* utilize fibrous vegetation via fermentative digestion in a forestomach and to a lesser extent in the caecum/proximal colon (Figure 2). The abdominal cavities of collected animals were opened and the GIT tied off above the foregut/esophagus junction and at the distal end of the rectum. The full GIT was stored on ice until dissection, within 5 hr. The tract was separated into four major compartments: the foregut, small intestine, caecum, and large intestine with rectum. Before removing contents, lengths along the midline of compartments were measured with a flexible tape measure; stretching was minimal on a wet dissection table. Tissue masses and content masses were measured to 0.1 g on a Mettler electronic balance. Average circumferences (including contents) of the small intestine, caecum, and large intestine were also determined via cloth measuring tape to 1 mm. For the small intestine, measurements were taken at 1, 2, 3, and 3.5 m along its length, with those for the caecum being its base, middle, and tip. Those for the large intestine were made 10 cm from the top, in the middle, and at its end. Samples of contents from foregut and rectum were taken to determine % DM in these compartments. These samples were also used to determine diets.

### 2.4 Skull and dental variation with age

Heads were taken from the individuals sampled for the GIT investigation and skinned and cleansed of flesh and brain. Dimensions of skull and teeth characteristics were obtained by following the procedures used by Lentle et al. (1998) for their studies of the tammar wallaby *Macropus eugenii*. Primarily, measurements were made with Vernier calipers in relation to the line of occlusion via projected planes, either parallel or at right angles to this line. Skull length was from the supraoccipital to the front of the premaxilla. Skull width was the maximum width across the zygomatic arch. Skull depth was taken from the highest point on the temporal bones to the lowest point on the mandible when the jaws were in occlusion. Skull volume was measured by wrapping each skull in fine plastic wrap and assessing the volume of water displaced by its emersion. Mandible length was measured from its back along the line of occlusion to the anterior tip of the procumbent incisor. Diastema + incisor distance for the lower jaw was from the foremost cheek tooth (premolar or molar) to the anterior tip of the procumbent incisor. Diastema length, upper jaw, was the distance from the foremost premolar/molar to the back of the posterior incisor. Incisor bite width was the maximum distance between the incisor wear facets across the upper incisor array. Incisor bite circumference relates to the size of the cropping bite and was measured as the extent of the active wear surfaces around the upper incisor array. These form when the two large lower procumbent incisors occlude against the semicircular array of 6 upper incisors during a bite. Molar/premolar wear surface estimates the active area of occlusion along the upper molar/premolar tooth rows during mastication. The area of wear on each active tooth was measured with Vernier calipers and summed. In the grazing kangaroos, the number of molar/premolar teeth in use varies with age (Hume, 1999).

### 2.5 Diets of free ranging kangaroos and vegetation structure in feeding areas

Vegetation surveys and diet determinations occurred in the summer and autumn of 1995, the initial one occurring concurrently with the sampling of kangaroos in February. Moderate summer rain, for this region (62 mm), had fallen in the preceding December and January and a flush of green grasses resulted. However, little rain fell from early February to mid‐May and pastures deteriorated; green, growing grasses were almost absent. Plant species occurrence and structure were measured in mid‐February (Hotel paddock) (*n* = 12 adults, *n* = 10 juveniles) and early May (Mating paddock) (*n* = 10 adults, *n* = 12 juveniles). The move to the Mating site, about 1.5‐km distance, allowed fecal pellets to be sampled from known, grazing animals; it was screened from the main road by a tree‐lined creek and kangaroos were somewhat habituated to researchers. At Hotel site, the relative cover and dry matter (DM) biomass of plant categories were determined using the multiple line transect procedures of Dawson and Ellis (1994); Dawson et al. (2004). At the Mating site, vegetation structure and DM biomass were obtained in collaboration with an ongoing study (Witte, 2002) and were calculated using the methods of Haydock and Shaw (1975). Due to dry conditions, the Mating site vegetation largely consisted of perennial grasses and chenopod shrubs and only these categories were examined.

Plants eaten by adult and juvenile *O. rufus* were determined from characteristic, microscopic, plant epidermal features in GIT contents or fecal pellets. This is feasible because, overall, epidermal tissue is poorly digested in arid zone plants (Dawson & Ellis, 1979). This, we also verified. Diet composition was estimated from the dried foregut and rectal samples (Hotel site) or from fecal samples from observed foraging individuals (Mating site) using the microscopic analysis methods and extensive plant reference slide collections (Dawson & Ellis, 1994).

### 2.6 Excretion rates, diet, and particle size distribution

In additional studies during 2001, adult females (5) and juvenile *O. rufus* (6) were examined in trials to investigate rates of digesta passage while they foraged for natural vegetation. The adults were tame kangaroos from the UNSW Animal Holding Facility, Cowan NSW. Prior to the trial, they spent 4‐month feeding in well‐vegetated enclosures in the vicinity of the Fowlers Gap Station homestead. The juveniles were obtained from the wild at the age of early emergence from the pouch, then raised on specialized milk, with this being reduced as their intake of natural vegetation in the enclosure increased. Weaning occurred ~3 weeks prior to the study. All kangaroos were placed in well‐vegetated individual pens a week before the start of the trial; additional feed was not provided. The pen sizes were approximately 5 × 8 m for juveniles and 5 × 10 m for adults; air temperature varied daily between 3 and 14℃ and shelter was provided.

Chromium and cobalt were used as the particulate and fluid markers, respectively, and were prepared using the method of Udén et al. (1980). The Cr was mordanted to the cell wall constituents of lucerne chaff and the Co was given as CoEDTA. The juveniles were fed approximately 1 g of mordanted Cr on a piece of white bread and 0.5 g of CoEDTA dissolved in 20 ml of milk was passed into the mouth via syringe. Such fluid is unlikely to bypass the foregut, as it can in juvenile ruminants. The foregut of macropodids is less specialized than in ruminants and a mechanism, especially in *O. rufus*, is in doubt (Hume, 1999). The next day, three adults ate 2 g of mordanted Cr and 1 g of CoEDTA soaked on bread; two adults did not ingest sufficient marker and were deleted from this aspect of the trial. Continuous monitoring occurred during the collection periods and fecal pellets were collected ~3 hr and 6 hr after dosing and then every 6 hr over 72 hr. The fecal pellets were analyzed for Cr and Co using a GBC 906AA atomic absorption spectrophotometer (GBC Scientific Equipment, 1989) and followed techniques also used in Munn and Dawson (2006). The concentration of the marker in mg/g fecal subsample was plotted against the cumulative time after the animals were fed the marker. The concentration is generally plotted in mg/g total fecal output but due to uncertainty with the collecting of the total amount of feces at night, the cumulative concentration was calculated per gram subsample. This assumes that the entire marker was excreted in the feces and that the animals' fecal outputs were relatively constant for each collection; these conditions appeared to be largely met.

Following the rate of excretion trials, fecal pellets were collected from each of the kangaroos and analyzed for diet proportions using the techniques of Dawson and Ellis (1994) and also the distribution of plant particle sizes. Particle sizes were determined after fecal pellets were teased apart in distilled water and then washed through sieves of two sizes, 500 and 125 μm. This gave particles greater than 500 μm and between 500 and 125 μm. Particles less than 125 μm were discarded; they mainly consisted of dust and broken microhairs (Dawson & Ellis, 1994). The size classes were centrifuged to constant volume to give proportions of size particles in the feces.

### 2.7 Statistical analysis

Statistical analyses were performed using *R* (R Core Team, 2020). Comparisons between adult and juvenile body sizes, and anatomical and physiological variables were made using *t* tests for variables that were lengths, circumferences, or masses, and beta regression using the R package “betareg” (Cribari‐Neto & Zeileis, 2010) for data that were proportions, with the significance of differences determined using the likelihood‐ratio test.

The relationships between gut content mass (foregut and total) and body mass were analyzed using linear mixed‐effects models, with a Gaussian error distribution and an identity link. Fixed covariates were stage (a categorical variable with two levels: juvenile and adult), and gut position (a categorial variable with two levels: foregut and total gut). To incorporate the dependency among measurements made on the same kangaroo, we used animal (ID) as a random intercept. We assumed that the association between body mass and gut contents mass followed the allometric relationship $y=aM_{i}^{b}$, where *y* is the mass of the gut component, $M_{i}$ is the body mass of individual *i*, and *a* and *b* are fitted parameters. The relationship was linearized prior to fitting by log–log transformation, giving $\log_{10}\left(y\right)=\log_{10}\left(a\right)+b\cdot \log_{10}\left(M_{i}\right)$. In total, four nested models were compared that represented increasing complexity in explanatory variables.

$$y_{\mathrm{ij}}∼\text{Normal}\left(\mu_{\mathrm{ij}},\sigma_{\mathrm{ij}}^{2}\right).$$

$$E\left(y_{\mathrm{ij}}\right)=\mu_{\mathrm{ij}}$$

(1) $$y_{\mathrm{ij}}={\text{stage}}_{\mathrm{ij}}+{\text{ID}}_{i}$$

(2) $$y_{\mathrm{ij}}={\text{component}}_{\mathrm{ij}}+{\text{ID}}_{i}$$

(3) $$y_{\mathrm{ij}}={\text{stage}}_{\mathrm{ij}}+{\text{component}}_{\mathrm{ij}}+{\text{ID}}_{i}$$

(4) $$y_{\mathrm{ij}}={\text{stage}}_{\mathrm{ij}}\times {\text{component}}_{\mathrm{ij}}+{\text{ID}}_{i}$$

$${\text{ID}}_{i}=N\left(0,\sigma^{2}\right).$$

where $y_{\mathrm{ij}}$ is the mass of the *j*th component or stage of individual *i*; and ID*_i_* is the random intercept, which is assumed to be normally distributed with mean 0 and variance $\sigma^{2}$.

The *R* package “lme4” (Bates et al., 2015) was used to fit the models and the model support was determined using the likelihood‐ratio test.

Nonlinear regression, using the *R* package “minpack.lm” (Elzhov et al., 2016), was used to estimate the parameters of the Bertalanffy growth function, which was used to determine the relationship between kangaroo age and bite characteristics (skull length and incisor bite circumference); molar wear area; and body mass.

Differences in the plant species in the diets of adults and juveniles were analyzed using Poisson generalized linear mixed‐effects models with a log link function. The Poisson distribution was suitable for the count data (number of plant particles) and the log link function ensured that the fitted values were positive. Factors tested included plant species (seven levels) and stage (two levels). For all fitted models that included plant species, to account for the correlation in the diet from an individual kangaroo, animal ID was included as a random intercept. The relationship between the frequency of plant particles in samples taken from the foregut and taken from the rectum, a Poisson generalized linear mixed‐effects model was again used. The fixed covariates of gut position (two levels: foregut, rectum) and stage (juvenile, adult) were included in the fitted models, and to account for correlation in the frequencies of plant particles from animals that were repeatedly measured, animal ID was included as a random intercept. Models were fitted using the R package lme4 (Bates et al., 2015) and model support was assessed using the likelihood‐ratio test.

The relationships between solute and particle excretion rates were nonlinear and variable transformation was unable to produce a linear relationship between the predictor variable (time since consumption of the marked food or liquid) and the rate of excretion of the marker. Consequently, the relationships were analyzed using generalized additive models (GAM) which can estimate smooth functional relationships between predictor variables and the response (Pedersen et al., 2019). For each response variable, two models were compared—one that estimated an overall smooth functional relationship between excretion rate and time (i.e., ignored differences between stages) and one model that estimated two smooth relationships—one for juveniles and a separate function for adults. The model with the most support was determined by comparison using AIC. For all models, the temporal correlation in excretion rates from an individual kangaroo was accounted for by including individual as a random factor. Model fitting was done with the R package “mgcv” (Wood, 2017), with model comparisons using the likelihood‐ratio test. The influence of feed characteristics on mean excretion rates of solutes and particles (from other published studies) were compared using *t* tests. Figures were produced using the package “ggplot2” (Wickham, 2016).

## 3 RESULTS

### 3.1 Body size and dimensions

**FIGURE 3:**
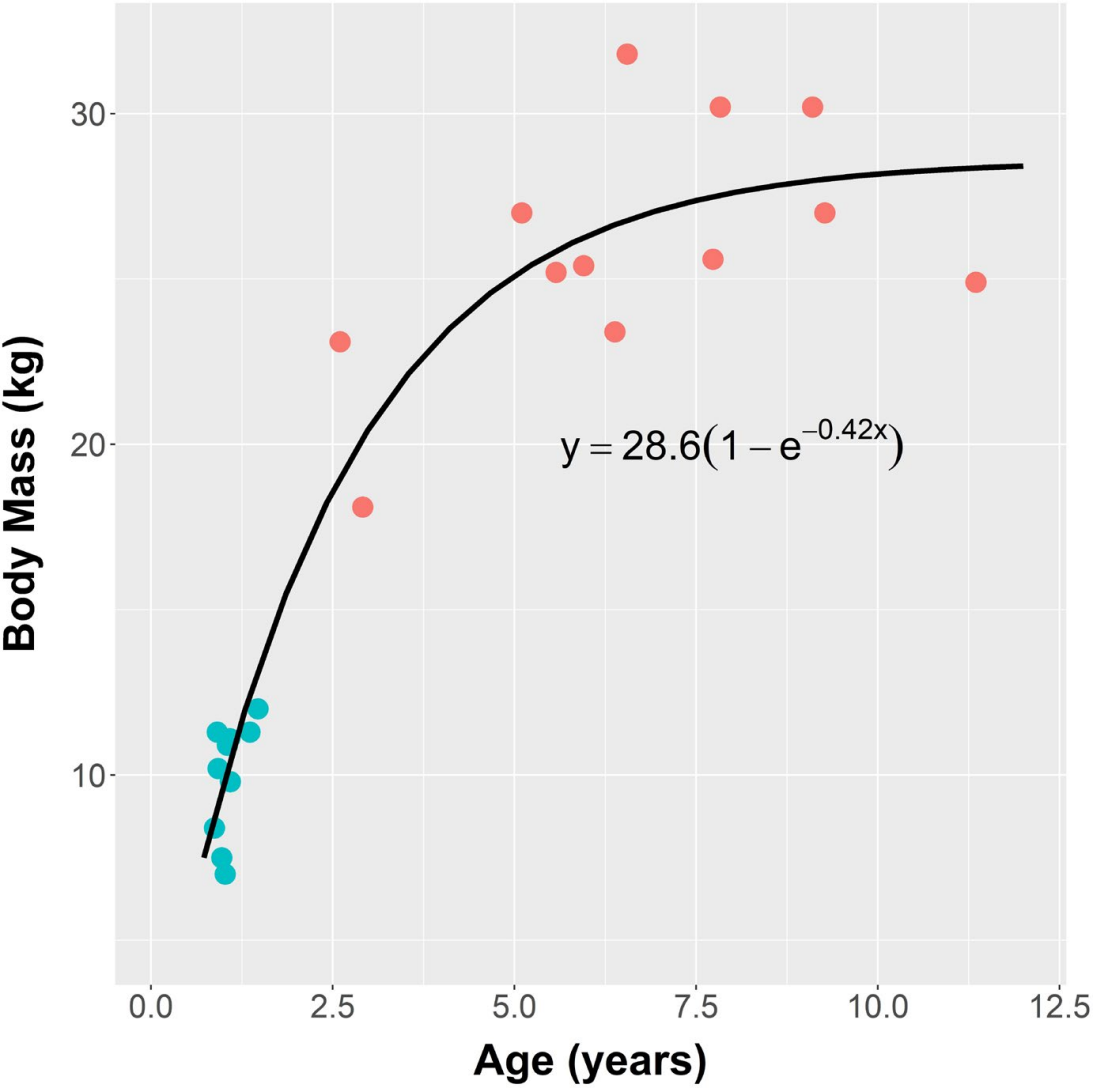
The relationship between age and body mass in *Osphranter rufus* from the weaning stage of juveniles (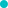) to maturity in adult females (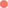). Age was determined from cleaned skulls using the molar index of Kirkpatrick (1965). The parameters of the fitted von Bertalanffy growth function, $m=m_{\text{max}}\left(1‐\exp\left(‐k\cdot \text{age}\right)\right)$, took the values $m_{\text{max}}$ = 28.6 (±1.08 *SE*) and *k* = 0.420 (±0.0458 *SE*)

Shown in Table 1 are the anatomical measurements from 12 adult females and 10 juveniles of weaning size, five females and five males. Mean body masses ± *SD* were 25.99 ± 3.70 kg for the adult female *O. rufus* and 9.95 ± 1.74 kg for the juveniles, that is, on average juvenile BM was 38% of that of the adult females. Ages derived using a molar index (Kirkpatrick, 1965) gave mean ages of adult females as 6.70 ± 2.56 years (range, 2.60–11.35 years) and for juveniles 1.07 ± 0.20 years (range, 0.87–1.47 years). At 1 year, the weaning of juveniles is usually complete (Dawson, 2012). These ages were comparable with those derived from limb proportion (*p* = 0.2 for adult females and 0.7 for juveniles; paired *t* tests). The pattern of variation in BM with age (Figure 3) shows rapid growth in the first 3 years of life. Thereafter, growth slows in the adult females as they approach a maximum BM of 28.6 kg.

**TABLE 1:** Body mass and dimensions together with assessed ages of adult females and juvenile *Osphranter rufus*

Dimension	Adult Female	Juvenile
Body mass (kg)	25.99 ± 3.70	9.95 ± 1.74***
Limb dimensions		
Foot length (mm)	310 ± 13	260 ± 16***
Leg length (mm)	499 ± 22	365 ± 21***
Arm length (mm)	221 ± 13	152 ± 10***
Total body length (mm)	1,586 ± 56	1,200 ± 61***
Age		
Arm/foot ratio (y)^a^	6.10 ± 2.21	1.10 ± 0.36***
Molar index (y)^b^	6.70 ± 2.56	1.07 ± 0.20***

Note

Values are means ± *SD*. *N* = 12 for adult females and 10 for juveniles.

^a^ Edwards (1990), Edwards et al. (1994).

^b^ From Table 7, Kirkpatrick (1965). Statistical difference (*t* test) between adults and juveniles.

*** *p* < 0.0001.

### 3.2 Gastrointestinal morphology

**FIGURE 4:**
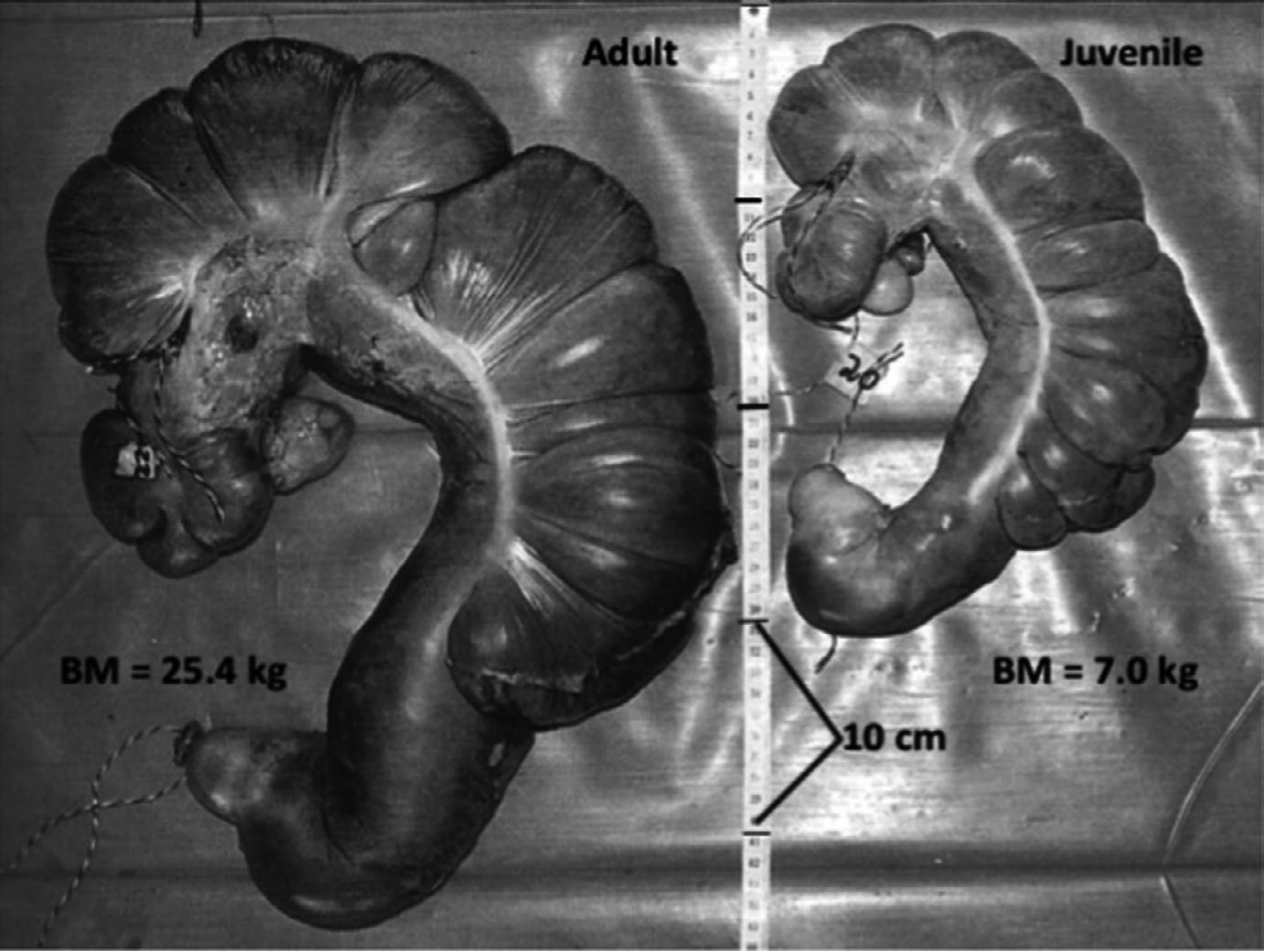
Full forestomach of adult and juvenile *Osphranter rufus*. Specimens obtained at end of morning feeding during good seasonal conditions at Fowlers Gap Arid Zone Research Station

**FIGURE 5:**
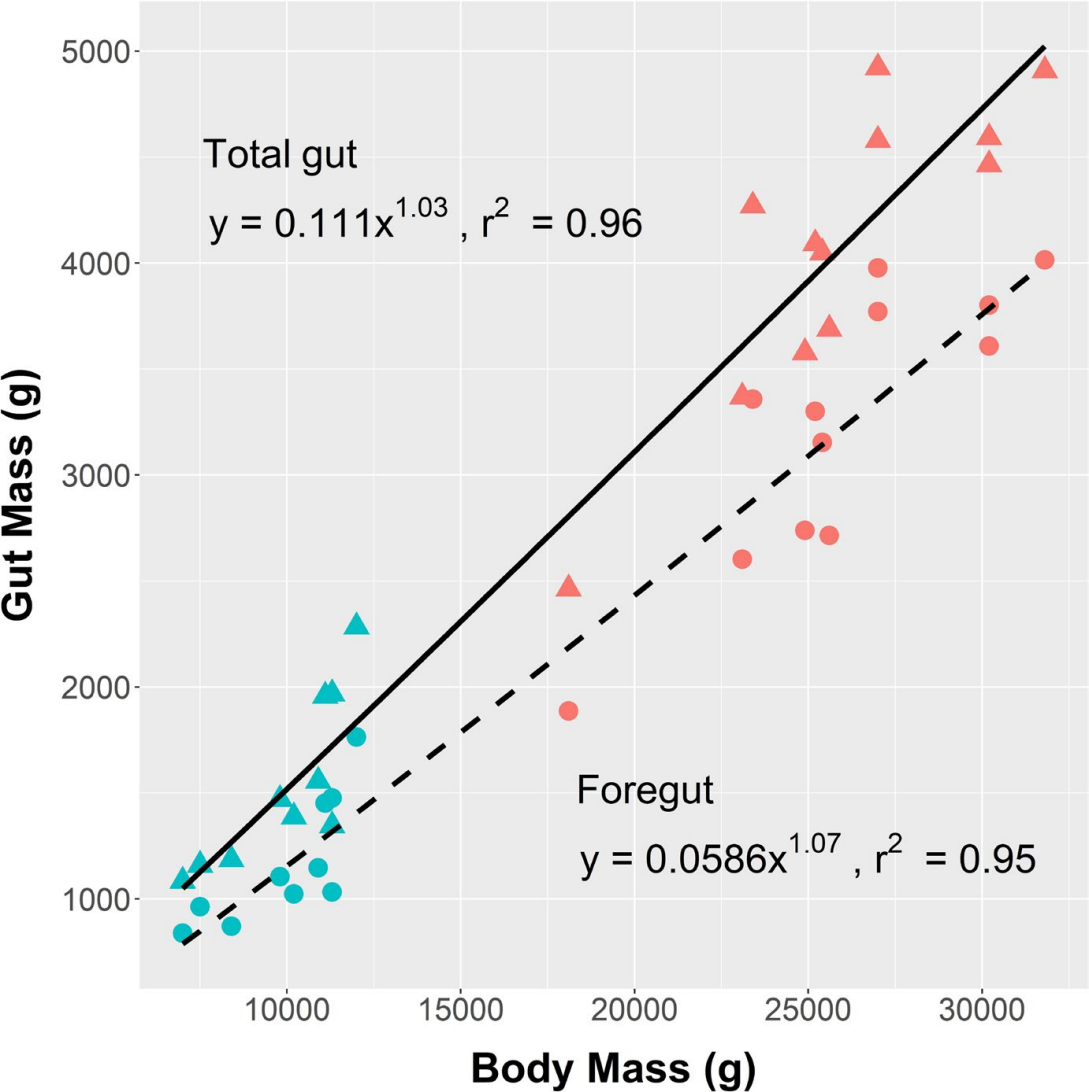
The relationships between the wet mass content of the foregut (——) and total gut (‐ ‐ ‐ ‐ ‐ ‐) and body mass in *Osphranter* *rufus* from the weaning stage of juveniles (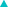, total; 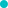, foregut) to maturity in adult females (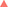,total; 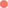, foregut)

The GIT dimensions and content characteristics for adults and juveniles taken near the end of morning feeding are shown in Table 2. The proportion of BM that the GIT (full) occupied was similar in adult females and juveniles; the full GIT of an adult was 20.2 ± 1.5% of BM as compared with 19.1 ± 2.4% for juveniles (*p* = 0.19, *t* test). The dominant size of the foregut in the tract of *O. rufus* is clear in Table 2, Figure 2. Figure 4 displays the characteristic form and relative size of the foregut in adult females and juveniles. Foreguts held more than three‐quarters of total GIT contents (WM). From the mean values in Table 2, adult *O. rufus* have foregut contents mass larger than juveniles, 79.1 ± 2.8% of the total GIT contents as against 76.0 ± 2.90% for juveniles (*p* = 0.017, *t* test). The linear mixed‐effects models that were used to determine the relationship between the factors stage (juvenile, adult) and gut component (foregut, total gut) indicated that the model that included the interaction between the factors received significantly more support than a model that did not include the interaction (*χ*² = 39.9, *p* < 0.001). This result implies that the relationship between gut contents mass and gut component depends on growth stage. Furthermore, when examining foreguts only, differences between foregut contents WM are explained by differences in BM alone, that is, the one regression line encompasses both age groups better than separate regressions for each age. Looking at total gut WM, the same pattern emerges; that is, one equation fits the data adequately and fit is not improved by including age group as a factor. The back‐transformed regression lines are plotted in Figure 5. No significant differences were seen between the age classes in the contributions of the small intestine, caecum, and large intestine/rectum to total GIT contents (Table 2).

**TABLE 2:** Gastrointestinal tract dimensions and content characteristics for adult female and juvenile *Macropus* *rufus* free ranging on rangeland in arid Australia

Gut region	Dimension	Adult female	Juveniles
Foregut	Foregut length (mm)	723 ± 66	496 ± 45***
	Empty mass (g)	482 ± 80	184 ± 35***
	Content, WM (g)	3,244 ± 652	1,168 ± 301***
	Cont., WM, % gut total	79 ± 3	76 ± 3*
	Content, DM (g)	488 ± 109	175 ± 41***
	% DM in contents	15.1 ± 2.0	15.2 ± 1.4n.s.
Small Intestine	Circumference (mm)	40 ± 6	31 ± 6**
	Length (mm)	5,408 ± 904	3,988 ± 715***
	Empty mass (g)	192 ± 54	101 ± 24***
	Content, WM (g)	348 ± 84	160 ± 51***
	Cont., WM, % gut total	8.7 ± 2.2	10.4 ± 2.2n.s.
Caecum	Circumference (mm)	88 ± 11	70 ± 12**
	Length (mm)	322 ± 31	233 ± 35***
	Empty mass (g)	37 ± 13	15 ± 5***
	Content, WM (g)	165 ± 47	63 ± 18***
	Cont., WM, % gut total	4.0 ± 0.8	4.1 ± 0.7n.s.
Large Intestine, Incl. Rectum	Circumference (mm)	59 ± 6	52 ± 19*
	Length (mm)	4,054 ± 611	2,891 ± 575***
	Empty mass (g)	173 ± 39	70 ± 18***
	Content, WM (g)	324 ± 56	150 ± 61***
	Cont., WM, % gut total	8.1 ± 1.7	9.5 ± 1.9n.s.
	Content, DM (g)	115 ± 42	57.0 ± 33***
	% DM in contents	35 ± 10	37 ± 13n.s.

Note

Values are means ± *SD*; *N* = 12 for adults; *N* = 10 for juveniles. DM = dry matter; WM = wet matter. Asterisks in juvenile column indicate levels of significant differences (*t* test for continuous variables, likelihood‐ratio test for proportions) from adult females.

n.s. indicates not significant, *p* > 0.05.

* *p* < 0.05

** *p* < 0.01

*** *p* < 0.001

### 3.3 Skull and dental variation with age

**FIGURE 6:**
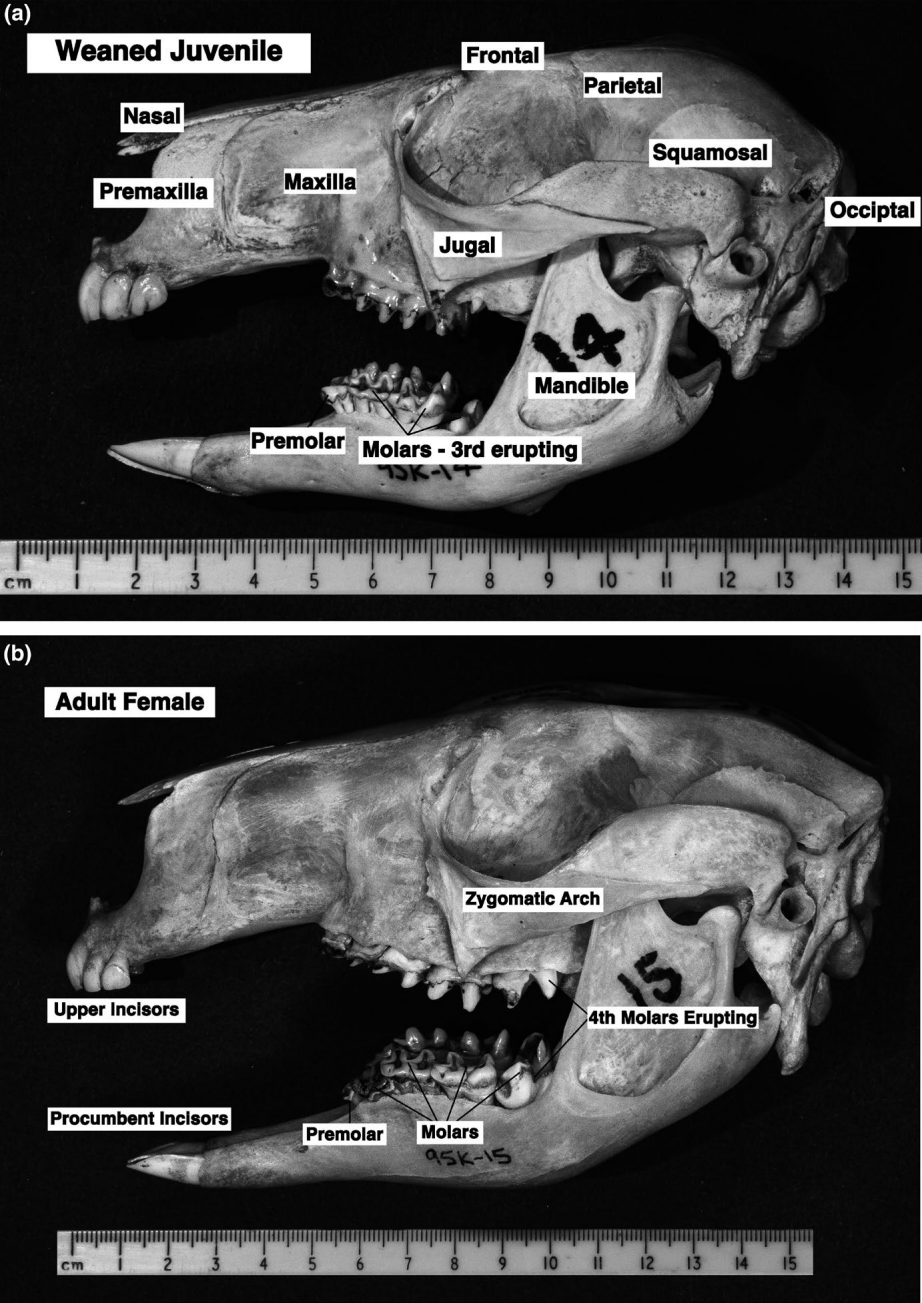
Skulls and teeth of *Osphranter rufus*. (a) Juvenile (1.09 year); (b) adult Female (6.38 year). Showing size and relative development of teeth. Dimensions of skull and teeth characteristics (Tables 8 and 9) were largely obtained by following the procedures used by Lentle et al. (1998)

**FIGURE 7:**
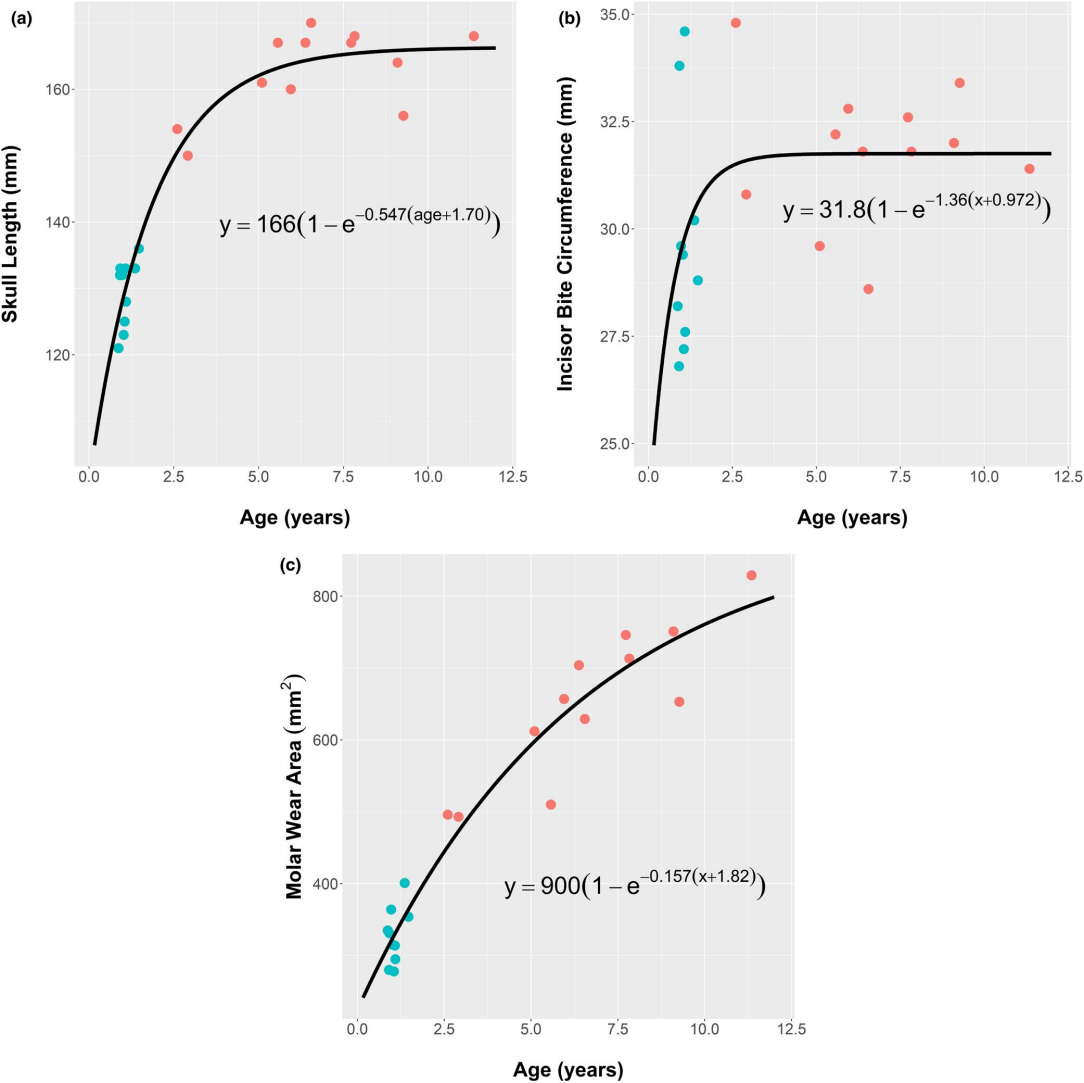
Variation of functional dental parameters from weaning to maturity in Osphranter rufus as measured from juveniles (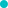) and adult females (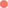). Ages were determined from cleaned skulls using the molar index of Kirkpatrick (1965). The parameters of the fitted von Bertalanffy growth functions, were for: (a) Skull length = 166 (±2.06 *SE*), *c* = −1.70 (±0.615 *SE*), and *k* = 0.548 (±0.131 *SE*); (b) Incisor bite circumference: = 31.8 (±0.700 *SE*), *c* = −0.972 (±3.39 *SE*), and *k* = 1.36 (±2.38 *SE*); (c) Molar wear area: = 900 (±122 *SE*), *c* = −1.82 (±0.678 *SE*), and *k* = 0.157 (±0.0593 *SE*)

As noted by Munn and Dawson (2003b), to maintain optimum growth newly weaned juvenile *O. rufus* need feed intakes approaching those of adult females, yet their mass is only 38% of an adult (Table 1). This quandary was further examined via an assessment of the forage harvesting apparatus of juveniles and adults, that is, their skulls and teeth. As anticipated, marked differences in skull and teeth characteristics and dimensions of the different age groups were seen (Table 3, Figure 6). However, patterns of difference are noteworthy. The relative skull size (volume) of juveniles is 52% of that of females; this pattern of relative increase is also seen for the area of molar/premolar active surfaces of juveniles. From a functional, dietary aspect, several features of the skull and the teeth stand out further (Table 3, Figure 6). Skull length of juveniles reached 80% of that of the adults and the bites of the incisors, that is, incisor bite circumference, were little different between the age classes. These disparities in functional dental features between juveniles and adults arise because of different rates of development within the skull (Figure 7a–c), as compared with the rate of increase in BM during growth from weaning to maturity (Figure 3). Bite depth, that is, the length of the sward that can be taken each bite, may be represented by diastema + incisor lengths; those of juveniles were also 82% of those of the adults (Table 3). Interestingly, this forward region of the skull occupies a set proportion of the skull length (32%) during its rapid elongation. (Figure 6).

**TABLE 3:** Skull and teeth characteristics of adult and juvenile *Macropus* *rufus* taken while feeding in rangeland of arid Australia (Fowlers Gap Arid Zone Research Station)

Dimension	Adult Female	Juvenile	Relative change
Index of molar progression (MI)^a^	3.16 ± 0.54	1.01 ± 0.21***	3.13
Age – MI (y)^a^	6.70 ± 2.56	1.07 ± 0.20***	6.26
Skull dimensions			
Skull length (mm)	163 ± 6	130 ± 5***	1.25
Skull width (mm)	86 ± 4	71 ± 2***	1.21
Skull depth (mm)	83 ± 5	64 ± 4***	1.30
Skull volume (cm^−3^)	478 ± 67	248 ± 24***	1.93
Mandible length (mm)	131 ± 5	100 ± 4***	1.31
Diastema + Incisors (mm)	52.2 ± 2.9	42.7 ± 2.3***	1.22
Diastema length, upper (mm)	42 ± 4	29 ± 2***	1.45
Incisor bite width (mm)	15.8 ± 1.2	13.1 ± 1.2***	1.21
Incisor bite circum. (mm)	31.8 ± 1.6	29.6 ± 2.7*	1.07
Molar/premolar wear surface (mm^2^)	649 ± 108	327 ± 39***	1.98

Note

Values are means ± *SD*. *N* = 12 for adults; *N* = 10 for juveniles.

Relative change is a measure of the increase in dimension from juvenile to adult.

^a^ Kirkpatrick (1965). Statistical difference (*t* test) between adults and juveniles.

* *p* < 0.05

*** *p* < 0.0001.

### 3.4 Diets of free‐ranging kangaroos and vegetation structure in feeding areas

Following summer rain diets at the Hotel site, determined from foregut contents in both the juveniles and adult females (Table 4), contained a predominance of grasses, these being near 90% of intake. Herbaceous forbs comprised 7% of intake in both groups. Overall, there was no difference in the broad diets selected by adults and juveniles in February (*χ*²(1) = 0.0012, *p* = 0.972). Their high selectivity for grasses is obvious when compared with the available vegetation that was predominantly chenopod shrubs, with some intermixed grasses at this time (Table 5), when ground cover by grasses was only 7 ± 5% of the area and contributed just 18% of total DM biomass. Grasses were mostly short perennial species such as *En*. *nigricans* (bottle washers), *Eragrostis setifolia* (neverfail), and *Tripogon loliiformis* (five‐minute grass) that were green from the recent rain. Round‐leaved chenopod shrubs, genera *Maireana* (blue bush) and *Sclerolaena* (poverty bush and copper burr), and flat‐leaved chenopod shrubs, genera *Atriplex* and *Rhagodia* (salt bushes), were almost absent from the diets, though they dominated the vegetation, contributing 41% and 30%, respectively, of DM biomass. The diets determined from rectal contents (Table 4) were not different to those derived from foregut (undigested) samples (*χ*²(1) = 0.0008, *p* = 0.978).

**TABLE 4:** Plant compositions of the foregut and rectal contents collected from adult female and juvenile *Osphranter* *rufus* foraging during moderate/good vegetation conditions in Australian arid rangelands

Sample site	Age class	Relative abundance of plant categories in the guts of adult and juvenile *Macropus* *rufus*, (%)						
		Grasses	Herbaceous forbs	Round‐leaved chenopods	Flat‐leaved chenopods	Malvaceous subshrubs	Browse	Other dicots
Foregut	Adult Female	89 ± 8.4	7 ± 5.2	1 ± 1.7	0.3 ± 1.0	1 ± 3.0	0	1 ± 3.4
	Juvenile	89 ± 9.7	7 ± 6.0	1 ± 2.4	0.7 ± 1.5	2 ± 4.8	0	0.7 ± 2.2
Rectal	Adult Female	91 ± 6.3	5 ± 4.9	1 ± 2.2	0.3 ± 1.1	3 ± 4.6	0	0
	Juvenile	89 ± 7.3	4 ± 4.0	2 ± 3.1	1 ± 2.6	3 ± 3.7	0.7 ± 1.5	0

Note

Values are means ± *SD*. *N* = 12 for adults; *N* = 10 for juveniles. Sampling 13–24 February 1995, Hotel Paddock.

Within each site/age class, ~4% of particles could not be assigned to a plant category.

**TABLE 5:** Contribution of major vegetation categories to total ground cover and biomass availability at the study sites at Fowlers Gap Research Station during 1995

Category	Hotel Paddock, February		Mating Paddock, April	
	Cover (%)	Biomass (g/m^2^)	Cover (%)	Biomass (g/m^2^)
Grass	7 ± 5	11 ± 6	5 ± 4	8 ± 5
Forbs	2 ± 3	7 ± 3	tr	2 ± 1
Round‐leaved Chenopod	6 ± 4	26 ± 6	4 ± 4	27 ± 5
Flat‐leaved Chenopod	4 ± 3	19 ± 5	2 ± 3	12 ± 3
Total	19 ± 12	63 ± 40	11 ± 5	50 ± 46

Note

Tr = trace, that is, <0,5%. Values are means ± S.D. Number of transects, each containing 100 points was for Hotel Paddock 10 and 15 for Mating Paddock.

NB. In February short, green grasses were common and provided 90% of grass biomass. In April, short grasses were much depleted and grass biomass came mostly as “medium” plants; detailed assessment of size classes was not made.

After February, no rain fell for over 100 days. Diet preference was again surveyed in late April, via fecal pellets at the comparable Mating site. Here, total ground covered by vegetation was only 11 ± 5% and plant DM biomass was 50 ± 46 g/m^−2^, excluding litter. Again, chenopod shrubs, mostly round‐leaved species, dominated, (Table 5). Ground cover by grasses was 5 ± 4% and they contributed 18% of total plant DM biomass; green leaf on grasses was sparse. Here, only the proportions of chenopod shrubs and grasses in the diet are given. (Table 6). In dry conditions, the diets of juveniles and adults diverged. Adult diets had moved to chenopod shrubs, 50 ± 10.9%, with grass declining to 38 ± 11.0%. However, juvenile diets still focused on grasses, 70 ± 8.8%, but chenopod shrub intake was up to 23 ± 8.2%. Overall, the proportion of grasses eaten relative to chenopod shrubs eaten differed significantly between the age groups, being 0.75 ± 0.14 for adults and 3.09 ± 0.47 for juveniles (*χ*²(2) = 66.1, *p* ≪ 0.0001).

**TABLE 6:** Percentage of major plant types in fecal pellets of adult and juvenile *Osphranter* *rufu*s at a time of lowered plant biomass, Mating Paddock April/May 1995

Age class	% Grasses	% Round‐ and flat‐leaved chenopods shrubs	% Unident. material
Adult	38 ± 11.0	50 ± 10.9	12 ± 3.4
Juvenile	70 ± 8.8	23 ± 8.2	7 ± 3.4

Note

Values are means ± *SD*. *N* = 10 adult females and 12 for juveniles.

### 3.5 Excretion rates

**FIGURE 8:**
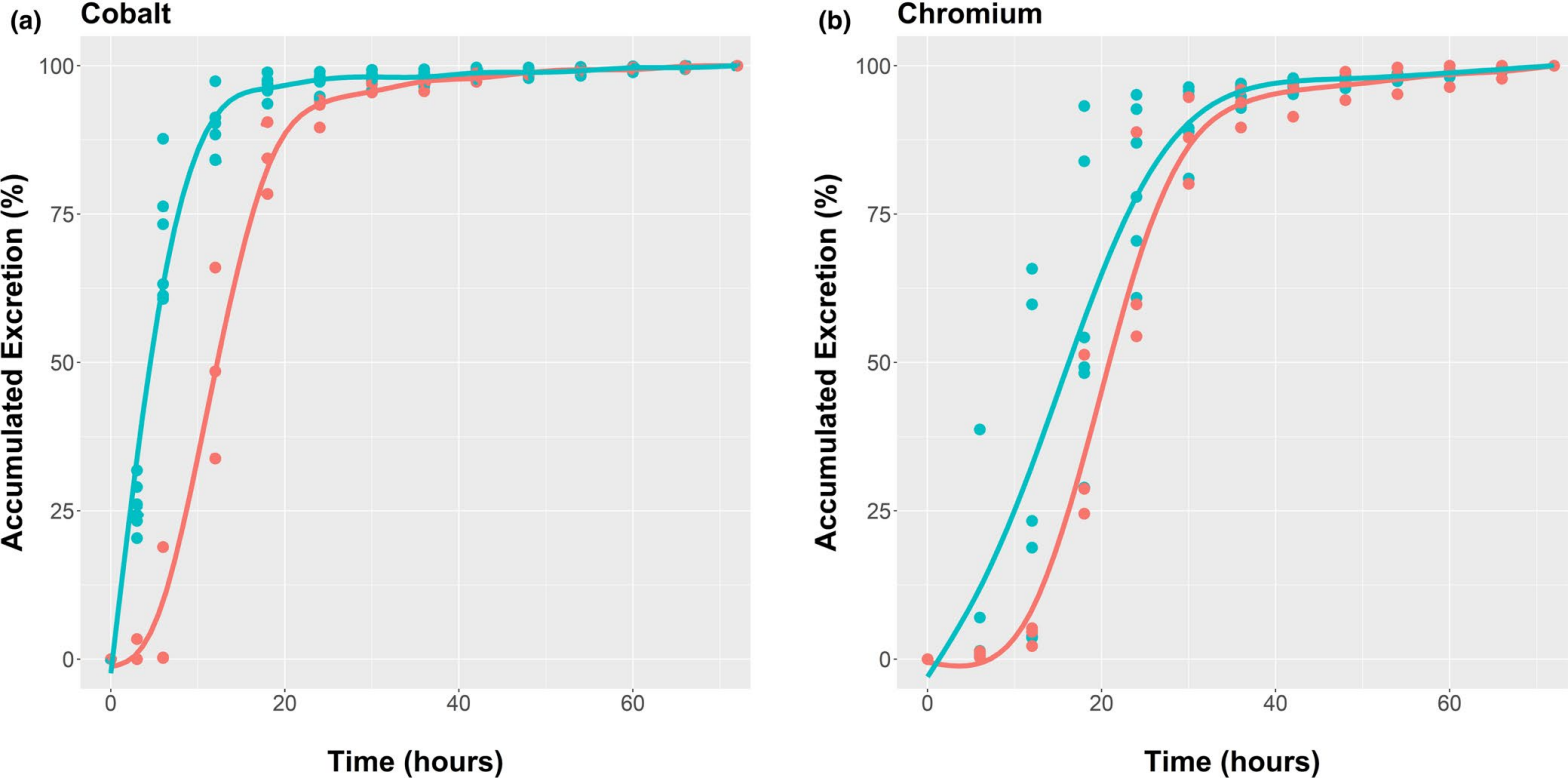
Cumulative excretion of (a) ingested fluid (cobalt) and (b) ingested particulate matter (chromium) by weaned juvenile (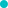) and adult female (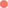) *Osphranter rufus* harvesting natural forage. The rate of excretion of the fluid differed significantly between the age groups. The difference in the rate of excretion of particulate matter between juveniles and adults was less pronounced than for fluid, but juveniles still had a faster excretion rate than adults

Cumulative excretion patterns are shown for both age classes in Figure 8a,b, respectively. Comparison of the models indicated greater support for the model examining solute passage rate that included the fixed effect stage, indicating significant differences between the excretion rates of solutes of juveniles and adults, (*χ*²(1) = 9,716.2, *p* ≪ 0.001), with juveniles having the faster rate. For particulate matter, the differences between juvenile and adult excretion rates were less pronounced, but juveniles still had a faster excretion rate than adults (*χ*²(1) = 1,895.5, *p* < 0.01). For comparative purposes, the 50% excretion values or mean retention times (MRT) are used commonly (Hume, 1999). Natural feeding MRTs are compared with laboratory studies (Table 7), where MRTs were derived for diets of chopped hay, with low or high fiber (Munn & Dawson, 2006). Animals feeding on green, natural forage have lower MRTs, notably so for the fluid fractions in the juveniles.

**TABLE 7:** Influence of feed characteristics on mean retention times for solute (Co) and particle (Cr) markers by adult and recently weaned juvenile red kangaroos following a pulse dose

Feed type	Food component	Mean retention time, (h)	
		Adult female	Juvenile, weaned
Natural rangeland feed	Solutes	12.0 ± 2.2	4.7 ± 0.5^*^
	Particles	21.0 ± 2.8	15.7 ± 5.2n.s.**^§^**
Chopped lucerne hay#	Solutes	15.7 ± 2.6	11.3 ± 2.4^*^
	Particles	28.6 ± 5.6	22.3 ± 6.6n.s.
Chopped oaten hay#	Solutes	16.3 ± 2.4	17.8 ± 2.8n.s.
	Particles	30.9 ± 3.8	30.1 ± 8.6n.s.

Note

Values are means ± *SD*. *N* = 5, except 3 for adult females eating natural feed. Mean body masses for animals foraging on natural feed were juveniles, 9.5 kg (±0.8 kg), and adults, 27.2 kg (range 25.7–30.3 kg). ^#^Values for lucerne and oaten hay, respectively, low and high‐fiber feeds, are from Munn and Dawson (2006). Asterisks in Juvenile column indicate levels of significant differences (*t* test) from adult females,*p < 0.05; n.s. indicates not significant, *p* > 0.05. (^§^Note that although the MRT for particulate matter excreted by juveniles and adults did not differ significantly, the more powerful GAM analysis indicated that the excretion rate by juveniles was faster than adults.)

### 3.6 Diet and particle size distribution associated with rate of excretion

The diets associated with the rate of excretion trial and the distribution of plant particle sizes excreted are shown in Table 8 and Table 9, respectively. The particle size categories were >500 µm and between 500 and 125 µm. There were no differences between adults and juveniles in the categories of plants eaten (*χ*²(6) = 7.48, *p* = 0.279) and no differences between adults and juveniles in the particle sizes in their feces for each food group (*χ*²(1) = 0.019, *p* = 0.890). However, there was strong evidence of an interaction between particle size and plant type (*χ*²(6) = 64.3, *p* ≪ 0.0001). There were significantly more particles of grass in the >500 µm category than in the 500–125 µm category. In contrast, there were significantly more particles of forb, malvaceous herbs, flat‐leaved chenopods, and round‐leaved chenopods in the 125–500 µm category than in the >500 µm category.

**TABLE 8:** The average frequencies (±*SD*) of major food categories in the feces of adult (*n* = 5) and weaned juvenile (*n* = 6) red kangaroos (*Osphranter rufus*)

Food group	Count	
	Adult	Juvenile (weaned)
Grass leaf	13.8 (±4.5)	25.0 (±10.3)
Grass stem	38.8 (±10.5)	13.7 (±4.1)
Grass seed	0.60 (±0.9)	0.33 (±0.5)
Forbs	4.0 (±1.0)	4.0 (±1.4)
Nonchenopod shrubs	2.6 (±2.2)	7.67 (±5.2)
Flat‐leaved chenopod	10.2 (±8.6)	16.0 (±7.5)
Round‐leaved chenopod	7.2 (±3.7)	11.0 (±5.1)
Unknown	22.6 (±6.0)	22.2 (±3.5)

**TABLE 9:** Volume, %, of the different particle sizes in the feces of adult female and juvenile *Osphranter* *rufus* in rate of passage trial in small yards with natural vegetation

Animal	Small %	Large %
Adult female (%)	33.5 ± 6.6	66.5 ± 6.6
Juvenile, weaned (%)	44.4 ± 6.1	55.6 ± 6.1

Note

Values are means ± *SD*. Small particles were <500–125> µm and large particles were >500 µm.

## 4 DISCUSSION

Juvenile *O. rufus* face severe nutritional challenges to their survival to adulthood. This study examined the mechanisms that may facilitate that survival in their harsh habitat. It has previously been shown that enhanced juvenile survival rates that maintain the species’ population structure are associated with periods of above‐average rainfall (Newsome, 1965). Significant challenges faced by recently weaned juvenile *O. rufus*, relate to the relatively high daily nutritional demands of growth and also body size allometry. Additional functional constraints relate to proportional gut size and fermentative digestion capacity. Despite *O. rufus* having a markedly different pattern of development from placental mammals (i.e., a long sojourn growing in a pouch), when they are weaned, they have functional equivalence to the weaned young of ruminant ungulates, which face similar issues with juvenile survival (Gaillard et al.,^1998^, ^2000^; Saether, 1997). Consequently, examination of *O. rufus* via a comparison of adult female and recently weaned juveniles can highlight adaptations and foraging strategies that are broadly applicable to all large herbivorous mammals.

The overall body sizes in the sample groups of wild *O. rufus* (Table 1 and Figure 3) agree with general growth patterns under moderate seasonal conditions (Dawson, 2012). Under such conditions, females may be sexually mature before they reach a BM of 20 kg, at around 3 years, with growth plateauing at 28–30 kg by 6–8 years (Figure 3). Female *O. rufus* exceeding 33–34 kg are uncommon except after a sequence of “good” seasons (Dawson, 2012). The juveniles had a mean age of 1.07 ± 0.20 years; sexually dimorphic divergence in BM between sexes (Dawson, 2012) had not begun.

Since the BM of juveniles at weaning is only 38% of the BM of adult females, the notion that they have daily energy needs close that of the adult female level (~93%) is challenging (Munn & Dawson, 2003b). This high nutrient requirement, while being partly due to the effect of metabolism varying with BM^0.75^, is mostly associated with the rapid growth of juveniles (Figure 3). The energy content of the tissue gained in growth was 5.3 kJ/g (Munn & Dawson, 2003b), which is equivalent to values reported for most young mammals. How do juvenile *O. rufus* achieve such dietary inputs relative to their BM, particularly in view of the constraints suggested for fermentative digestion (Demment & Van Soest, 1985; Parra, 1978)?

Examination of the GIT of *O. rufus* gave little insight into this question. Apart from size, GITs of adults and juveniles of *O. rufus* differ little in structure and apparently in operation (Table 2; Figures 4, 5; Griffiths & Barton, 1966). The basic structure of the GIT (Figure 2) has functional similarities to the rumen of the bovid ungulates but is not as complex (Hume, 1999). The large foregut with many haustrations (Figure 2) dominates the GIT tract and is the major site of the fermentative digestion of their grazing diet. Additionally, it has a distal gastric pouch (Figure 2; Figure 4) that can be likened to the abomasum of the bovid rumen complex, in that it also secretes pepsin and hydrochloric acid for enzymatic digestion (Griffiths & Barton, 1966). As with other kangaroos (Freudenberger, 1992), the intestines and hindguts of *O. rufus* are also, structurally and functionally similar to those of ruminants such as goats, that is, some subsidiary fermentative digestion also occurs in the caecum/colon region. The functional similarity of the GITs of the age classes is supported by the dry matter contents of digesta not differing through their GITs (Table 2), being 15% in the foreguts and 35%–37% in the rectums.

The similarity in structure and operation between the GITs of adult and juvenile *O. rufus* provides a key insight into how juveniles meet their additional nutritional needs. Regression analysis (Figure 5) showed that with both GIT content and foregut content, one regression line encompasses both age groups better than separate regressions for each age. If gut capacities, as indicated by contents at the end of morning feeding, seem not to explain how juveniles meet their high DEIs, other factors must be involved, likely related to diet selection. If foregut capacity is constrained, the amount of feed processed is dependent on the ease of its digestibility, that is processing time (Robbins, 1993). The diets of wild adults and juveniles *O. rufus* were assessed in good (Table 4) and poor (Table 6) seasonal conditions. In the good season, grasses, mostly green, dominated the diets (~90%) of all *O. rufus*; relative to the available biomass (Table 5), grasses were preferentially selected by both age classes (Table 4). In the subsequent dry conditions, grasses were still selected from the available biomass. However, while they provided most of the juvenile diet (70%), adult female intakes moved to 50% chenopod shrubs, with grasses comprising only 38% of their diet. This pattern in the adult *O. rufus* complies with long‐term studies (Dawson & Ellis, 1994) and may reflect their GIT plasticity (Munn & Dawson, 2006). However, the juvenile data give little further insight into how they achieve their high DEIs.

There are broadly two ways to achieve additional throughput and greater digestion of feed intake. One approach, seen in the ruminant ungulates, involves additional processing of digesta to reduce particle size and increase digesta particle surface area, enabling use of more abundant, refractory vegetation (Bjorndal et al., 1990; Perez‐Barberia & Gordon, 1998). The other option is more selective feeding that focuses on more easily digestible plants. Macropodid marsupials such as *O. rufus* do not ruminate and seemingly have no mechanism for the delay of digesta. Particle size distribution in kangaroos appears to be determined by initial oral processing and remains subsequently unchanged as the digesta passes through the GIT (Freudenberger, 1992). Examination of fecal pellets associated with rate of digesta passage studies (Table 9) supports this pattern. No significant differences occurred between adults and juveniles in the particle sizes in their feces for each food group. However, evidence for interactions between particle size and plant types warrants further investigation.

The rates at which vegetation is processed can reveal its basic characteristics. Rates of digesta passage of *O. rufus* foraging naturally on rangeland (Figure 8a,b) gave overall mean retention times (MRT) for *O. rufus* that were less than seen for low‐fiber feed in the laboratory (Table 7), markedly so for naturally feeding juveniles. That MRTs in large mammalian herbivores scale with BM^0.25^ have been suggested from allometric modeling, but this is based on the proviso that digestibilities are equivalent and this is not necessarily so (see review of Clauss et al., 2013). In our study, the MRTs of natural feeding kangaroos varied with BM^0.81^, *R* ^2^ = 0.95, for particles and BM^0.78^, *R* ^2^ = 0.96 for solutes. Thus, the juvenile *O. rufus* with the shorter MRTs have higher forage digestion levels than the adult females.

An understanding of these results and their consequences for digestion in *O. rufus* requires insight into the variability in digestibility among rangeland plants, including their parts and especially their stages of maturity (Short et al., 1974). The cellular contents of forage plants, such as sugars, lipids starch, soluble proteins, and nonprotein nitrogenous compounds, are rapidly digested by ruminants (Short et al., 1974; Van Soest, 1967) and marsupials such as kangaroos (Dellow & Hume, 1982). However, cell wall components (CWC), that is, fiber, are more variably digested, with some, such as lignin, being largely indigestible. Kangaroos such as *O. rufus* are grass specialists (Table 4; Dawson & Ellis, 1994), and in the context of this study, the characteristics of grasses are most significant. Young grass suspended in a nylon bag in a goat rumen by Short et al. (1974) was 75% digested within 4 hr and almost completely digested within 32 hr. Conversely, mature, dry grass was just 15% digested within 4 hr and only reached 57% digestion after 168 hr. Types of herbage (forbs) showed similar patterns but with more variation. Although, in this study, there was no delineation of plant groups into species or growth form, it was clear from the rates of passage of digesta that juvenile *O. rufus* focused on the most digestible components of vegetation (Table 6), thus providing more nutrients for a given foregut capacity.

Are there further mechanisms that help the juveniles achieve their high nutrient needs (Munn & Dawson, 2003b)? That the foregut of *O. rufus* juveniles is a smaller proportion of the total GIT contents than in adult females, suggests that they necessarily focus on forage that can be rapidly digested, such as young, green grasses, or herbage. For this to be achieved, feed harvesting must be proportionally increased by juveniles, but how is this managed? The primary parameters involved in harvesting are feeding time, bite rate, and bite size. While a detailed assessment of feeding time allocation by juveniles relative to adult *O. rufus* is not yet available, differences are likely. Foraging patterns of subadult (15–17 kg) and adult female *O. rufus* in the wild indicated that the smaller animals were more selective feeders (Watson & Dawson, 1993); they spent less time chewing and more time searching than the adults. Additionally, examination of skulls and teeth (Figures 6 and 7a‐c; Table 3) showed the potential for relatively enhanced harvesting capabilities in juvenile *O. rufus*.

A central feature of these data was the relative skull size (volume) of the recently weaned juvenile *O. rufus*, which reached 52% of adult size, when juvenile BM was only 38% of adult BM. Broadly, the harvesting parameters for bite size in mammalian herbivores are bite circumference, bite depth and the size, and characteristics of processing surfaces of teeth. The patterns of change for these parameters during growth in *O. rufus* are shown in Figure 7a‐c. Notable, is the relative size of the incisor bite of juveniles, as judged from the incisor bite widths and circumferences (Table 3); the mean incisor bite circumference of juveniles was 93% of the adult mean. Similarly, bite depth, that is, the length of the grass sward taken with each bite, may be inferred from the relationship of mandible or diastema lengths to skull lengths. Given the manner in which the macropodid jaw elongates with age (Lentle et al., 1998), diastema + incisor length can provide a proxy for bite depth; the juveniles have lengths that are 69% of adult lengths. Thus, the cropping capabilities of the two age classes are closer than indicated even by proportional head size.

The primary function of the complex molar/premolar array, “the dental mill” (Figure 6), in kangaroos is to break down fibrous grasses for more rapid fermentative digestion in the forestomach. In juveniles, the molar/premolar wear surface area is proportionally enlarged but only as with skull size. While this gives juveniles a dental mill that is enlarged relative to body size, they are disadvantaged relative to digestible energy needs. This reinforces the view that they must process less fibrous vegetation than adults do, to achieve their energy needs.

In summary, obtaining sufficient nutrients to achieve maturity is a challenging ecophysiological issue for recently weaned juvenile *O. rufus*, as it is generally among mammalian herbivores. Nutritional needs are much elevated relative to their BM due to body size allometry and, especially, for growth; however, foregut capacity is constrained. Field measurements show major gut structures of juveniles are directly related to BM, as are those of adults, that is, ~BM^1.0^. The diets of both juvenile and adult *O. rufus* largely focus on grasses. However, rates of digesta excretion obtained from naturally foraging *O. rufus* suggest that juveniles with notably faster excretion times (compared with adults) are preferentially selecting young, green grasses, or herbage that can be rapidly digested. That the foregut in juveniles occupies proportionally less of the total gut also suggests a greater quantity of forage may be harvested. Characteristics of the skulls of juveniles relative to those of adults revealed how this can occur. Firstly, while juvenile BM is ~38% of adult BM, the skull size (volume) is 52% of that of adults. Additionally, in juveniles the skull lengthens proportionally faster, especially in the forward portion containing the dentition. Of note, their incisor bite size approaches that of adult females. However, the area of wear on molar surfaces did not increase faster than overall skull size, again pointing to the selection of more digestible vegetation (less fibrous) forage by juveniles.

Overall, juvenile *O. rufus* have relatively large nutritional needs that initially seem insurmountable. However, they improve their capacity by diet selection, having a relatively bigger dental harvesting apparatus and a relatively faster GIT turnover rate. Even with these adaptations, the juveniles of *O. rufus* generally require extended periods with above‐average rainfall to provide substantial population recruitment. However, Frith and Sharman (1964) report some survival to adulthood even during poor environmental conditions via slower growth rates of juveniles. These patterns of responses by juvenile *O. rufus* to the metabolic challenges of growing quickly in a fluctuating rangeland environment are likely mirrored in the life of other young large mammalian herbivores. Also, they indicate in detail why, in times of environmental stress, juvenile mortality dominates the structure of herbivore populations.

## CONFLICT OF INTEREST

Authors have no conflicts of interest.

## AUTHOR CONTRIBUTIONS

**Terence J. Dawson:** Conceptualization (lead); Data curation (lead); Funding acquisition (lead); Investigation (lead); Methodology (lead); Project administration (lead); Resources (lead); Supervision (lead); Validation (equal); Writing‐original draft (lead); Writing‐review & editing (equal). **Melinda A. Norton:** Conceptualization (supporting); Data curation (supporting); Formal analysis (supporting); Investigation (supporting); Methodology (supporting); Writing‐original draft (supporting); Writing‐review & editing (supporting). **Suzette Rodoreda:** Conceptualization (supporting); Data curation (supporting); Formal analysis (supporting); Investigation (supporting); Project administration (supporting); Visualization (supporting); Writing‐original draft (supporting); Writing‐review & editing (supporting). **Sarah K. Abbott:** Data curation (supporting); Investigation (supporting); Methodology (supporting); Project administration (supporting); Writing‐review & editing (supporting). **Steven R. McLeod:** Data curation (equal); Formal analysis (equal); Funding acquisition (supporting); Investigation (supporting); Software (equal); Validation (equal); Writing‐review & editing (equal).

## Data Availability

The original base data gathered for this study are archived in Dryad: (https://doi.org/10.5061/dryad.6hdr7sr11).
